# Effects of Plasma Treatment on the Strength of Bonding to Ceramic Surfaces in Orthodontics—A Comprehensive Review

**DOI:** 10.3390/bioengineering10111323

**Published:** 2023-11-16

**Authors:** Elizabeth Gershater, Olivia Griswold, Brooke E. Talsania, Yu Zhang, Chun-Hsi Chung, Zhong Zheng, Chenshuang Li

**Affiliations:** 1 Division of Orthodontics, College of Dental Medicine, Columbia University, New York, NY 10032, USA; 2 Department of Orthodontics, School of Dental Medicine, University of Pennsylvania, Philadelphia, PA 19104, USA; 3 School of Dental Medicine, University of Pennsylvania, Philadelphia, PA 19104, USA; 4 Department of Preventive and Restorative Sciences, School of Dental Medicine, University of Pennsylvania, Philadelphia, PA 19104, USA; 5 David Geffen School of Medicine, University of California, Los Angeles, Los Angeles, CA 90095, USA

**Keywords:** plasma, surface modification, shear bond strength, zirconia, porcelain, ceramic

## Abstract

Over the past several decades, orthodontic treatment has been increasingly sought out by adults, many of whom have undergone restorative dental procedures that cover enamel. Because the characteristics of restorative materials differ from those of enamel, typical bonding techniques do not yield excellent restoration–bracket bonding strengths. Plasma treatment is an emerging surface treatment that could potentially improve bonding properties. The purpose of this paper is to evaluate currently available studies assessing the effect of plasma treatment on the shear bond strength (SBS) and failure mode of resin cement/composite on the surface of ceramic materials. PubMed and Google Scholar databases were searched for relevant studies, which were categorized by restorative material and plasma treatment types that were evaluated. It was determined that cold atmospheric plasma (CAP) treatment using helium and H_2_O gas was effective at raising the SBS of feldspathic porcelain to a bonding agent, while CAP treatment using helium gas might also be a potential treatment method for zirconia and other types of ceramics. More importantly, CAP treatment using helium has the potential for being carried out chairside due to its non-toxicity, low temperature, and short treatment time. However, because all the studies were conducted in vitro and not tested in an orthodontic setting, further research must be conducted to ascertain the effectiveness of specific plasma treatments in comparison to current orthodontic bonding treatments *in vivo*.

## 1. Introduction

The number of adult patients seeking orthodontic care has increased dramatically in the past decades, a trend currently sweeping the globe that shows little sign of reversing [1,2,3]. For example, 30% of patients receiving orthodontic treatment in the United States in 2016 were adults, compared to only 4.37% in 1960 [2]. Similarly, in the United Kingdom, three-quarters of orthodontists surveyed by the British Orthodontic Society in 2019 reported treating more adults than they had before [4]. Importantly, in comparison to adolescent patients, adults have a higher rate of having undergone restorative dental procedures involving the use of dental materials (such as veneers, inlays, onlays, and crowns) that cover or replace the enamel before receiving orthodontic treatment [1,5].

Thanks to the fast-growing field of dental materials, various ceramic materials are currently utilized in restorative and aesthetic dentistry [5,6,7]. For instance, porcelain (namely feldspathic ceramics) has been increasingly used due to its aesthetic qualities [6,7,8,9], and glass ceramics (such as lithium disilicate) are also becoming more popular [9,10]. In addition, zirconia, especially yttria-stabilized tetragonal zirconia polycrystal (Y-TZP) and its variants, is being increasingly applied in dentistry because of its superior mechanical properties and chemical inertness [10]. However, since the composition and characteristics of these ceramic materials differ from native enamel, the adhesion of orthodontic brackets and attachments to these restorations likewise differs from their adhesion to the natural tooth surface [1,5]. These differences in adhesion result in a high debonding rate of brackets and attachments from the ceramic surface that substantially affects orthodontic treatment [11].

Many techniques have been developed to resolve the debonding problem with the aim of enhancing the porosity and roughness of the surface of these ceramic materials and thus improving the bonding strength for orthodontic purposes [5]. Some well-known examples include the use of hydrofluoric acid (HF) etching, the application of silane treatment, and sandblasting (air-abrasion) with aluminum oxide particles [1,12]. Currently, sandblasting is generally the most frequently used technique [1], though, HF etching with subsequent silane treatment is considered to produce the best surface conditioning of feldspathic and glass ceramic restorations [13]. However, because HF can be harmful and particularly aggressive to soft tissues, with the potential to cause oral soft tissue necrosis [1,5], crucial precautions must be taken when using HF intraorally [11,12,14,15]. In addition, zirconia is not amenable to surface treatment by acids, including HF [13,14,16,17]. For example, Mehmeti et al. demonstrated that HF application could weaken the surface structure of zirconia (as well as lithium disilicate) and thus may actually compromise these ceramics structurally [15,18]. Likewise, sandblasting has also been shown to cause structural damage to ceramic materials such as zirconia, including creating surface and subsurface cracks [16,17,19,20,21]. A number of newly emerging surface treatment strategies, such as lasers and plasma, are currently being investigated to overcome these hurdles [18,19,22,23]. Notably, current research on plasma surface treatments spans a wide variety of techniques, from radiofrequency plasma spraying [24] to plasma-enhanced chemical vapor deposition [25] to cold atmospheric plasma (CAP) [26]. At the same time, many different gases, including argon and helium, have been applied in plasma treatments [23,26]. There are multiple review articles that extensively discuss the mechanism of the plasma treatments on surface modification, as well as their influence on the surface chemical composition of the substance materials [27,28,29,30]. However, there is still a need to compare and evaluate the effects of these different plasma-based techniques to determine which ones are practical for clinical chairside application and most effective at providing adequate bonding strength, specifically in the context of orthodontic applications.

Currently, when evaluating the adhesion of brackets to ceramic restorations, researchers are principally interested in two parameters: shear bond strength (SBS) and failure mode [5]. SBS testing is considered a substitute for evaluating the forces exerted by the jaw during mastication and a reliable method for quantifying the degree of adhesion [31,32]. It is important to note that a low SBS may lead to an increased chance of debonding, while an excessively high SBS can be detrimental to the tooth surface or restorative material and is, therefore, also undesirable [31,33]. A 1975 study testing a variety of metal brackets attached to enamel reported that a tensile bond strength of approximately 4.9 MPa has been suggested as sufficient for clinical success, but there is no suggestion on the SBS values [34]. With the improvement of dental materials, the use of clinically common bonding agents such as the primer Transbond XT [35,36] provides SBS values of 15–33 MPa [37], which can be considered a clinically accepted standard for orthodontics.

Failure mode, on the other hand, describes how debonding between the adhesive and the substrate occurs [38]. Specifically, “adhesive failure” refers to debonding at the interface of the resin cement and either the bracket or the surface of the tooth/restoration, “cohesive failure” refers to debonding within the resin cement/composite so that some resin cement remains on both the bracket and the tooth/restoration, and “mixed failure” refers to debonding characterized by both adhesive and cohesive failure [38,39,40]. Generally, a tendency toward cohesive failure rather than adhesive failure indicates that the bonding forces between the substrate–adhesive interface are more robust than the bonding forces within the adhesive itself; thus, a tendency for cohesive failure indicates good bonding strength between the resin cement and bracket/enamel/restoration and is therefore more desirable for orthodontic treatment [38,41,42].

By comparing and evaluating the SBS and failure mode results from currently available publications assessing the efficacy of plasma treatment on ceramic surfaces, we intend to gain insight into the potency of plasma treatment in enhancing the adhesion between orthodontic brackets and the surfaces of ceramic restorative materials. Accordingly, this review article could pave the way for establishing new clinically practice guidelines for ceramic surface conditioning in orthodontics.

## 2. Materials and Methods

The following keywords were used for the literature search in MEDLINE (PubMed) and Google Scholar: (ceramic and plasma), (zirconia and plasma). Only original studies were included in the analysis, and we excluded reviews, case reports, opinions, and letters to editors. In addition, the references of the included articles were screened, and the relevant articles were hand-searched. Subsequently, relevant information, including the type of ceramic, plasma type and conditions, bonding reagents, the SBS, and the failure mode, were extracted from each included article. As significant variations were noticed in the SBS values of the control groups across the studies, the “% of Control SBS” was calculated by dividing the mean value of the SBS of each experimental group by the mean value of the SBS of its respective control group to represent the extent of SBS changes caused by plasma treatment.

## 3. Feldspathic Porcelain

Feldspathic porcelain is one of the most commonly used restorative materials in aesthetic dentistry [6,8,43]. As an all-ceramic material composed of aluminosilicates that may contain other metals such as calcium, potassium, and sodium [6], feldspathic porcelain presents excellent translucency and closely resembles natural enamel [44]. Despite its good aesthetic qualities, feldspathic porcelain has a flexural strength of 60–120 MPa [6,45], indicating it is brittle and prone to chipping [46,47] and thus may complicate orthodontic treatment.

### 3.1. Cold Atmospheric Plasma (CAP)

CAP, also known as non-thermal plasma (NTP) [48], is the most commonly tested plasma treatment method for feldspathic porcelain (Table 1). For example, Adımcı et al. utilized CAP in combination with silane adhesive to treat feldspathic porcelain [49]. In their study, the gas used for the plasma treatment was not specified; thus, the chairside safety of the treatment cannot be assessed. However, the CAP application did not involve high temperatures, had a relatively short treatment time of 90 s, and yielded an SBS of 43.16 MPa, which is about 20% higher than that of the control (silane primer treated w/o CAP) and is moderately above the acceptable SBS range of 15–33 MPa [37,49]. Regarding failure mode, although the silane primer control and CAP + silane adhesive treatment groups had a relatively similar distribution of adhesive, cohesive, and mixed failures, the CAP-treated group was nevertheless characterized by a greater mixed failure value [49].

All of the studies that specified which gas was used for CAP treatment of feldspathic porcelain used helium gas in combination with other gases [50,51,52] (Table 1). These helium-based plasma treatments yielded an SBS value of 11.5–32.4 MPa, with the highest SBS achieved by the helium/hexamethyldisiloxane (HMDSO)/benzene CAP treatment [50,51,52]. Unlike the controls (no primers), whose failure modes were entirely or overwhelmingly characterized by adhesive failure [50,51,52], helium-based CAP treatments’ failure modes had a higher incidence of cohesive and/or mixed failure mode and a lower incidence of adhesive failure mode [50,51,52]. Noticeably, these helium-based plasma treatments only require 60 s or less treatment time and involve low temperatures [50,51,52], which is preferable for clinical settings. However, even though helium is a non-toxic gas, most of the other gases used, such as benzene, HMDSO, and triethylene glycol dimethacrylate (TEGDMA) [50,51,52], are either toxic or very dangerous to handle [54,55,56]. For instance, benzene is a known carcinogen [54]. Thus, safety concerns are a massive obstacle to using these plasma treatments on humans.

On the other hand, Han et al. established a CAP treatment method using helium with vaporized, non-toxic distilled water [51]. This treatment yielded an SBS value of 26.3 MPa (which is 30% greater than the SBS value of the untreated control and is within the clinically acceptable range for orthodontic treatment) [37,51]. In addition, 60% of the plasma-treated samples underwent adhesive failure and 40% underwent either cohesive or mixed failure, compared to 100% adhesive failure in the control group [51], indicating that the plasma treatment resulted in stronger bonding between the adhesive and the brackets/feldspathic ceramic and therefore would be a good addition to a protocol for attaching brackets to feldspathic ceramic restorations. More importantly, the method was designed with intraoral usage in mind: the treatment time was short (30 s), and plasma was applied with a hand-held ceramic pencil-type plasma torch [51]. In addition, the torch was situated 0.5 cm from the treated surface, the flow rate was set to 2 L/min, and power was generated at 5.15 W and 15 kHz [51]. The CAP treatment is low-temperature and the application of plasma can be performed by hand instead of using machinery such as a reactor, suggesting that it may be a viable surface treatment for intraoral feldspathic ceramic restorations during orthodontic treatment.

### 3.2. Plasma Etching

In addition to CAP, etching with plasma followed by silane application was initially developed as a feldspathic ceramic surface treatment by Çökeliler et al. [53]. While a non-toxic gas, oxygen, was used for plasma etching, and high temperatures were avoided in this method, the current technology is not ready for chairside use due to the long treatment time of 15 min [53]. Moreover, this treatment resulted in an overly large SBS value of 72.83 MPa [37,53], indicating a bonding strength that is similar to the flexural strength of feldspathic porcelain and far exceeds what is normally needed for orthodontic purposes and thus may cause damage to the restoration during the debonding process. Undoubtedly, modification of this plasma etching treatment is required to optimize it for clinical use.

### 3.3. Summary

Of all the different types of plasma activation that have been tested on feldspathic porcelain, CAP with helium and water can yield an SBS value similar to that of HF-based bonding treatment, and only a short treatment time and no toxic gas is necessary, thus holding a great deal of potential for treating the surface of feldspathic porcelain during orthodontic bonding. However, the influence of porcelain surface aging and saliva contamination on the bonding strength achieved by plasma treatment still needs to be investigated, as well as the treatment’s potential toxicity to local oral tissues and the body overall. Moreover, although previous studies showed that HF etching combined with adhesives could result in an SBS of up to 22.01 MPa on the treated feldspathic porcelain surface [12,57], all available studies assessing the effect of plasma on feldspathic porcelain SBS used untreated rather than HF-treated controls (Table 1). Thus, whether plasma activation is superior to HF treatment for feldspathic porcelain should also be assessed via direct comparison.

## 4. Zirconia

Zirconia, the oxidized form of zirconium, is classified as a polycrystalline ceramic [6]. Other materials, such as calcia, ceria, magnesia, and, most notably, yttria, may be added to stabilize the tetragonal and cubic phases of zirconia at ambient temperatures [6]. While zirconia is generally characterized by its strength and resistance to damage [10], Y-TZP is especially recognized for its ability to withstand wear and tear within the mouth [6]. Since its aesthetic qualities are inferior to those of other restorative materials (such as feldspathic ceramics and glass ceramics), Y-TZP has been more widely studied and used in posterior tooth restorations than anterior ones [58,59,60]. Noticeably, previous studies suggested that zirconia is inherently unamenable to treatment with HF [16,17], so establishing an effective plasma treatment for chairside surface conditioning of zirconia ceramic restorations for orthodontic purposes is especially important. Moreover, exposure to water can cause low-temperature degradation of tetragonal zirconia. Damage, such as microcracks, can develop in the surface of zirconia restorative materials [61,62], so further research on how the moist oral environment can affect the bonding strength of zirconia is also needed.

### 4.1. Cold Atmospheric Plasma (CAP)

#### 4.1.1. CAP with Argon

Non-toxic argon is the most widely used gas for CAP treatment on zirconia, alone or in combination with oxygen. The major differences among the currently available studies are the plasma treatment time, primer usage, cement/composite type, and zirconia brand, summarized in Table 2.

##### Argon-Alone CAP

Argon-alone CAP treatment of zirconia can be carried out in less than 1 min; however, these short-term treatments generally resulted in a low SBS value below the acceptable range [63,64,65] (Table 2). On the other hand, prolonged argon-alone CAP treatment time may be detrimental to bonding strength. For example, in comparison with their responsive controls, 1 min argon-alone CAP activation with G-CEM LinkACE resin cement application on zirconia lowered the SBS values [66], and 30 min argon-alone CAP activation with Panavia F2.0 resin cement application reduced the SBS value of the zirconia surface by 35.38% compared to its control [23]. Based on the available literature, argon-alone CAP did not alter the failure mode of the treated zirconia surface [23,63,64,67]. Taken together, despite the treatment times that ranged from short (30 s) to long (30 min) periods, previous studies did not suggest that argon-alone CAP could provide good bonding strength between zirconia and the bonding agent.

**Table 2:** SBS and failure mode of CAP-treated zirconia ceramic. CAP: cold atmospheric plasma; SBS: shear bond strength. For failure mode, A: adhesive failure; C: cohesive failure; M: mixed failure.

Plasma Gas(es)	Zirconia Type	Primer (Y/N)	Subgroup Differences		SBS (MPa)	% of Control SBS	Failure Mode (A/C/M)	Control Failure Mode (A/C/M)	Ref.
Argon	Zirmon^®^ specimens (Kuwotech, Gwangju, Republic of Korea)	N	Panavia F2.0 resin cement	exposure to atmosphere 0 h	4.22 ± 0.99	64.62%	0/0/100	0/0/100	[23]
				exposure to atmosphere 24 h	5.19 ± 0.92	79.48%	0/0/100		
				exposure to atmosphere 36 h	6.05 ± 1.70	92.65%	0/0/100		
				exposure to atmosphere 72 h	6.98 ± 1.96	106.89%	0/0/100		
		Y	RelyX U200 resin cement	exposure to atmosphere 0 h	5.26 ± 0.83	170.78%	0/0/100	90/0/10	
				exposure to atmosphere 24 h	5.08 ± 1.15	164.94%	0/0/100		
				exposure to atmosphere 36 h	4.24 ± 0.76	137.66%	0/0/100		
				exposure to atmosphere 72 h	2.71 ± 1.19	87.99%	100/0/0		
Argon	Katana blocks (Kuraray Noritake, Tokyo, Japan)	N	plasma treated for 10 s	not stored in water	6.9	153.33%	100/0/0	100/0/0	[63]
				stored in water	2.9	152.63%	100/0/0		
			plasma treated for 30 s	not stored in water	7.7	171.11%	100/0/0		
				stored in water	2.9	152.63%	100/0/0		
	ZirCAD blocks (Ivoclar Vivadent, Schaan, Liechtenstein)		plasma treated for 10 s	not stored in water	11.4	165.22%	100/0/0	100/0/0	
				stored in water	3	136.36%	100/0/0		
			plasma treated for 30 s	not stored in water	10.7	155.07%	100/0/0		
				stored in water	2.8	127.27%	100/0/0		
	Katana blocks (Kuraray Noritake, Tokyo, Japan)	Y	plasma treated for 10 s	not stored in water	9.7	190.20%	100/0/0	100/0/0	
				stored in water	4.9	175.00%	100/0/0		
			plasma treated for 30 s	not stored in water	11.9	425.00%	100/0/0		
				stored in water	4.6	164.29%	100/0/0		
	ZirCAD blocks (Ivoclar Vivadent, Schaan, Liechtenstein)		plasma treated for 10 s	not stored in water	11.9	156.58%	100/0/0	100/0/0	
				stored in water	5.2	260.00%	100/0/0		
			plasma treated for 30 s	not stored in water	9.6	126.32%	100/0/0		
				stored in water	5.5	275.00%	100/0/0		
Argon	KZ-3YF type AC powder (KCM, Nagoya, Japan)	N	not colored		6.46 ± 0.372	152.36%	100/0/0	100/0/0	[64]
			colored with molybdenium chloride		5.89 ± 0.237	138.92%	100/0/0	100/0/0	
			colored with chromium chloride		7.29 ± 1.082	171.93%	100/0/0	100/0/0	
Argon	Katana plates (Kuraray Noritake, Tokyo, Japan)	N	not stored in water		10.3 ± 4.7	105.10%	-	-	[65]
			stored in water		7.4 ± 4.0	79.57%			
	Lava plates (3M ESPE, St. Paul, MN, USA)		not stored in water		12.3 ± 3.8	93.18%			
			stored in water		7.4 ± 4.0	137.04%			
Argon	InCoris Maxi-S blocks (Sirona, Charlotte, NC, USA)	Y	Futurabond U adhesive	BifixSE luting composite, not stored in water, not thermocycled	25.4 ± 6.1	104.10%	100/0/0	100/0/0	[67]
				BifixSE luting composite, stored in water, thermocycled	1.5 ± 1.2	- (0 MPa for control)	100/0/0	100/0/0	
				BifixQM luting composite, not stored in water, not thermocycled	23.9 ± 4.9	114.35%	100/0/0	100/0/0	
				BifixQM luting composite, stored in water, thermocycled	9.8 ± 5.2	98.00%	100/0/0	100/0/0	
			Futurabond M^+^ adhesive	BifixSE luting composite, not stored in water, not thermocycled	23.1 ± 6.6	126.23%	100/0/0	100/0/0	
				BifixSE luting composite, stored in water, thermocycled	5.0 ± 9.2	1666.67%	100/0/0	100/0/0	
				BifixQM luting composite, not stored in water, not thermocycled	27.3 ± 4.8	131.25%	100/0/0	100/0/0	
				BifixQM luting composite, stored in water, thermocycled	14.6 ± 3.5	124.79%	100/0/0	100/0/0	
			Futurabond M^+^ + DC-Activator adhesive	BifixSE luting composite, not stored in water, not thermocycled	22.6 ± 8.6	96.58%	100/0/0	100/0/0	
				BifixSE luting composite, stored in water, thermocycled	1.9 ± 1.2	- (0 MPa for control)	100/0/0	100/0/0	
				BifixQM luting composite, not stored in water, not thermocycled	16.7 ± 3.0	74.22%	100/0/0	100/0/0	
				BifixQM luting composite, stored in water, thermocycled	11.8 ± 4.0	142.17%	100/0/0	100/0/0	
Argon	LUXEN cubes (DentalMax, Seoul, Republic of Korea)	Y	G-CEM LinkACE resin cement	not thermocycled	19.99 ± 4.67	82.81%	0/0/100	50/0/50	[66]
				thermocycled	6.66 ± 0.81	62.54%	0/0/100	50/0/50	
			RelyX U200 resin cement	not thermocycled	12.62 ± 3.82	120.31%	10/0/90	10/0/90	
				thermocycled	4.14 ± 0.61	47.92%	10/0/90	10/0/90	
Argon, oxygen	Cercon Smart Ceramics plates (Degudent, Madrid, Spain)	Y	treated for 2 min		24.34 ± 4.95	164.57%	40/40/20	80/20/0	[68]
			treated for 5 min		27.89 ± 3.31	188.57%	30/30/40	80/20/0	
Helium	Katana specimens (Kuraray Noritake, Tokyo, Japan)	Y	-		16.6 ± 0.64	164.36%	-	-	[69]
Helium	TT-MT (A1) cylindrical specimens (Upcera, Pforzheim, Germany)	Y	not thermocycled		23.00 ± 0.79	166.06%	10/0/90	80/0/20	[26]
			thermocycled		19.92 ± 0.87	190.26%	40/0/60	80/0/20	
Not specified	CEREC InCoris ZI specimens (Dentsply Sirona, Charlotte, NC, USA)	N	not thermocycled		4.4 ± 0.3	157.14%	70/0/30	100/0/0	[70]
			thermocycled		3.1 ± 0.3	155.00%	90/0/10	100/0/0	
		Y	not thermocycled		10.0 ± 1.8	142.86%	20/0/80	40/0/60	
			thermocycled		7.2 ± 0.7	160.00%	40/0/60	60/0/40	
Not specified	IPS e.max ZirCAD blocks (Ivoclar Vivadent, Schaan, Liechtenstein)	N	-		20.22 ± 1.76	180.05%	-	-	[71]

It is still questionable whether adding primer after argon-alone CAP treatment meaningfully increases SBS. For instance, two different groups reported that argon-alone CAP + primer treatment on zirconia surfaces could provide orthodontically acceptable SBS values (ranging from 12.6 to 27.3 MPa); however, the SBS values of the plasma-activated zirconia surfaces were not statistically significantly different or were even lower than those of the respective controls [66,67], which casts doubt on whether these CAP treatments indeed improved the dental materials’ bonding strength. Two other investigation groups reported that although argon-alone CAP + primer treatment could increase SBS values, the resulting SBS was still largely below the range acceptable for orthodontic treatment [23,65]. Thus, the combination of argon-alone CAP and primer application does not ensure an increased SBS in the acceptable range. Further research is needed to clarify the best combination of argon CAP and bonding reagent(s) for clinical usage.

It is also worth noting the variation in the types of zirconia used in the available studies (Table 2). Although most of these types of zirconia consistently presented inadequate SBS values (less than 10 MPa) when treated with plasma [23,63,64,65,66,67], there was nevertheless notable variation across these studies (Table 2). For example, under non-water storage and non-thermocycling conditions, Zirmon^®^ specimens (Kuwotech, Gwangju, Republic of Korea) presented the lowest SBS value (2.71 MPa) after argon-alone CAP treatment [23]; in contrast, InCoris Maxi-S blocks (Sirona, Charlotte, NC, USA) and LUXEN cubes (DentalMax, Seoul, Republic of Korea) demonstrated much higher SBS values (27.3 MPa and 19.99 MPa, respectively) [66,67]. The usage of strong bonding reagents may be an explanation of the high SBS exhibited by argon-alone CAP-treated InCoris Maxi-S blocks (Sirona) and LUXEN cubes (DentalMax) zirconia [66,67], as treated LUXEN cubes (DentalMax) show distinctly different SBS values when bonded with G-GEM LinkACE resin cement compared to RelyX U200 resin cement [66]. However, variation in SBS values was also observed when evaluating different types of zirconia treated with the same bonding reagents. For example, when bonded with primer and RelyX U200 resin cement, Zirmon^®^ specimens (Kuwotech) had an average SBS of 2.71 MPa [23], while LUXEN cubes (DentalMax) had an average SBS of 10.49 MPa [66]. More significantly, under non-water storage and non-thermocycling conditions, argon-alone CAP treatment could improve the SBS of Zirmon^®^ (Kuwotech) specimens (when bonded with RelyX U200 resin cement) [23], ZirCAD blocks (Ivoclar Vivadent, Schaan, Liechtenstein) [63], and KZ-3YF type AC powder (KCM, Nagoya, Japan) [64], but did not affect or even reduced the SBS of InCoris Maxi-S blocks (Sirona) [67]. Thus, both bonding reagents and zirconia types are important factors in the post-CAP treatment bonding strength.

Nevertheless, there is notable inconsistency amongst the findings of the studies. Katana blocks (Kuraray Noritake, Tokyo, Japan) without primer were used by two groups [63,65]; Negreiros et al. claimed that argon-alone CAP treatment could improve the SBS by 50–70% (increasing with plasma treatment time) [63], but de Mendonça et al. concluded that CAP treatment does not affect the SBS [65]. With such considerable variation in the currently available publications, further confirmation of test results is needed.

Importantly, many procedural aspects of argon-alone CAP treatment (i.e., distance of the plasma source from the surface, gas flow rate, and water storage) varied among studies, making it difficult to ascertain the exact reason why different SBS values were achieved. On the other hand, it is also challenging to determine the weighted contribution of each factor when similar SBS values were obtained from studies with different protocols. For example, of all the studies that omitted primer, Park et al. and Negreiros et al. are the only ones that reported SBS values more than 50% higher than the control [63,64]. However, there are significant differences between the two studies: Park et al. used a gas flow rate of 10 L per minute and did not store specimens in water, while Negreiros et al. used a rate of 1 L per minute and stored specimens in water for 24 h after treatment [63,64]. It is important to emphasize that storing specimens in water or artificial saliva at 37 °C is a valuable tool for mimicking intraoral conditions and may yield more representative SBS values than simply storing specimens in dry conditions. However, studies under such conditions have not yet been performed. Thus, in-depth investigations are needed to better understand how specific procedural factors can contribute to CAP treatment impacting the SBS of zirconia.

##### Combined Argon and Oxygen CAP

Unlike the argon-alone CAP treatments that did not notably increase the SBS of zirconia materials [23,63,64,65,66,67], combined argon and oxygen CAP conditioning of a zirconia surface yielded orthodontically acceptable SBS values (24.35 MPa resulting from a 2 min treatment and 27.89 MPa resulting from a 5 min treatment) [68] (Table 2). Meanwhile, the argon/oxygen CAP-treated zirconia surface displayed moderately higher incidences of cohesive and mixed failure and lower incidences of adhesive failure in the two plasma-treated groups compared to the control [68], further indicating that the plasma treatment strengthened the bonding between the resin cement and zirconia. It is worth noting that although the 5 min argon/oxygen CAP treatment led to a slightly higher SBS value than the 2 min one [68], it may be too long for chairside use, possibly making the 2 min treatment a better option for use in the clinic.

#### 4.1.2. Helium CAP

Helium has also been explored to as a potential gas to use in CAP treatment of zirconia ceramic [26,69] (Table 2). In two studies, CAP was conducted with a hand-held piece to apply plasma 10 mm from the ceramic surface, and primer was applied to the zirconia surface after the plasma treatment [26,69]. Ito et al. reported an SBS of 16.6 MPa, while Ye et al. reported an SBS of 23.00 MPa after helium CAP treatment; both SBS values are within the orthodontically acceptable range and are at least 64% higher than the SBS of their respective primer-only control groups [26,69]. More excitingly, when the zirconia specimens were thermocycled to mimic the intraoral environment, the helium CAP treatment still resulted in an increased SBS of 19.92 MPa, 90% higher than the control [26]. In addition, Ye et al. reported a significant shift in the distribution of failure mode post-helium-CAP treatment [26] (Table 2). Specifically, the control group had an 80% incidence of adhesive and a 20% incidence of mixed failure modes, which remained unchanged after 24 h storage in water [26]. In contrast, the helium CAP-treated group had a 10% incidence of adhesive and a 90% incidence of mixed failure mode; after water storage, the incidences of adhesive and mixed failure modes were 40% and 60%, respectively [26]. This tendency of the experimental group towards mixed failure even after water storage [28] indicates that the helium plasma treatment enhances the strength of the bonding between the resin cement and zirconia. Furthermore, helium CAP treatment of zirconia specimens only took 30–90 s [26,69], making it highly amenable for chairside use in orthodontic practices.

Notably, the Katana zirconia tested in the study of Ito et al. [69] has also been explored for argon-alone CAP treatment [63,65] (Table 2). These previous studies suggested that Katana zirconia had a better response to helium CAP treatment. However, it will be necessary to conduct side-by-side studies comparing different types of CAP treatment on the same zirconia material before a definite conclusion can be drawn.

#### 4.1.3. CAP with Unspecified Gas

We also found two studies in which zirconia was treated with CAP but the gas used was not specified [70,71] (Table 2). Specifically, Altuntas et al. reported that a 90 s CAP treatment induced ~50% SBS increase, which was still below the acceptable range, accompanied by a rise in the incidence of mixed failure [70]. On the other hand, Mahrous et al. found that an 80 s treatment led to an ~80% SBS increase and thus fell into the acceptable range, while no evaluation of failure mode was presented [71]. Regardless of the results of these two studies, it is impossible to further compare these studies with others without specific information on the type(s) of gas used for CAP.

### 4.2. Plasma-Enhanced Chemical Vapor Deposition (PECVD)

Multiple studies have also explored the use of plasma-enhanced chemical vapor deposition (PECVD) for treating zirconia surfaces using combinations of argon with oxygen, HMDSO, hydrogen, sulfur hexafluoride, benzene, and tetramethylsilane (TMS) (Table 3). Overall, these studies showed that PECVD using argon, oxygen, and HMDSO could significantly reduce the SBS of Y-TZP [25,72], while PECVD using argon, hydrogen, TMS, or benzene could improve the SBS of zirconia [73], although these improved SBS values do not satisfy the clinical standard for orthodontic treatment. On the other hand, the PECVD treatment using argon and silane yielded an acceptable SBS of the zirconia surface without noticeably altering the incidence of failure modes [74]. Importantly, all these studies require the use of a chamber and toxic gases and employ an overly long treatment time of 5 to 10 min [25,54,55,72,73,74,75,76,77], which rules out the intraoral use of current PECVD modification surface modification methods for zirconia ceramics.

### 4.3. Fluorination

Fluorination with sulfur hexafluoride (SF6) is another plasma surface treatment that has been applied to different types of zirconia, resulting in consistently increased SBS values ranging from 26.3 to 37.3 MPa [78,79,80] (Table 4). However, it is interesting to note that the studies, including two that tested the same type of zirconia, did not agree on the differences in the distribution of failure modes [78,79,80] (Table 4). Meanwhile, although fluorination achieved clinically acceptable SBS values, this technique seems to have significant drawbacks: not only were the treatment times relatively long (2 and 5 min), but also the fluorination reactions were carried out in a reactor and conducted at high temperatures that could approach 100 °C, which would not be possible to replicate intraorally [78,79,80]. Moreover, SF6 can cause tissue damage and, in the presence of electric discharge, break down into HF molecules, which, as stated previously, can also be harmful [76]. Thus, although fluorination does result in significant improvements in bonding strength, this type of plasma treatment is not currently feasible for intraoral surface modification of zirconia restorative materials.

### 4.4. Magnetron Sputtering

Magnetron sputtering has also been evaluated on zirconia ceramics (Table 5). Karakış et al. carried out an argon-alone radiofrequency magnetron sputtering treatment for 2.5, 15, and 20 min, followed by primer application, and yielded SBS values higher than 20 MPa [81]. Unfortunately, the 2.5 min treatment only improved the SBS by 10%, while the 15 and 20 min are too long for chairside use despite yielding higher increases in the SBS. Moreover, short-treatment-time argon-alone radiofrequency magnetron sputtering treatment without primer sometimes even reduced the SBS of zirconia [81], further confirming that argon-alone radiofrequency magnetron sputtering treatment may not be an efficient surface treatment strategy for zirconia. In contrast, using argon and oxygen gas for reactive or radiofrequency magnetron sputtering, followed by silane application, could significantly raise the SBS of zirconia [82,83]. However, magnetron sputtering reactions were carried out in chambers with high pressure and a long treatment time (30 to 60 min), making this type of plasma treatment nonviable for chairside application.

### 4.5. Plasma Etching

A study by El-Shrkawy et al. used oxygen, a non-toxic gas, for a treatment combining plasma etching and primer [84] (Table 6). This yielded an SBS of 17.8 MPa, more than 190% greater than the primer-treated control [84]. The control group had a 30% incidence of adhesive failure and 70% incidence of mixed failure, in contrast with the experimental group, which had a 30% incidence of cohesive failure and 70% incidence of mixed failure; despite the low incidence of adhesive failure in both groups, these data nevertheless indicate that the oxygen etching treatment enhanced the bonding strength of the resin cement to the zirconia [84]. However, a treatment time was not specified in that study, making it difficult to assess whether this treatment can be used in a clinical setting [84]. This is the only currently available publication reporting the effect of plasma etching on zirconia, so further studies are needed to verify these findings.

### 4.6. Silica Coating

The study by El-Shrkawy et al. mentioned in the preceding section also evaluated silica coating treatments using argon gas followed by primer application [84], and found that this conditioning yielded an SBS of 19.6 MPa, more than 200% greater than the primer-treated control (Table 6). In addition, the silica coating treatment resulted in a 70% incidence of cohesive failure and 30% incidence of mixed failure, indicating that the silica coating treatment improved the bonding strength [84]. However, the silica coating technique used by the study requires equipment that precludes intraoral use and therefore cannot be used for chairside treatment [84].

### 4.7. Radiofrequency Plasma Spraying

Oxygen and HMDSO have been used in radiofrequency plasma spraying on two types of zirconia (Table 6), yielding SBS values below the accepted range but significantly higher than those of the non-plasma control [24]. In addition to this treatment’s poor outcome, this method cannot be replicated intraorally due to its use of toxic HMDSO and the RF plasma spraying technique involved [24].

### 4.8. Glow-Discharge Plasma

Egoshi et al. reported a glow-discharge plasma treatment of zirconia in which the gas was not specified [85] (Table 6). Certain elements of this technique (such as the treatment being carried out in a vacuum) prevent it from being replicated intraorally [85], thereby making it unsuitable for chairside use. However, it is worth noting that glow-discharge plasma can potentially significantly increase the SBS value of zirconia ceramics, and the type of resin used determines whether the SBS value is orthodontically acceptable [85]. In addition, although there were generally no differences in failure mode incidence between the control and plasma-treated, non-primer luting composite of Panavia V5 groups, the plasma-treated Clearfil SA Luting Plus cement group had a notably higher incidence of mixed failure mode compared to its control group after 24 h water storage [85], again indicating the importance of bonding reagent selection.

### 4.9. Plasma Irradiation

An irradiation plasma treatment on zirconia was used by Noro et al., which was not particularly successful [86] (Table 6); the resulting SBS values were lower than or similar to those of the controls [86]. Moreover, the irradiation procedure cannot be carried out intraorally.

### 4.10. Summary

These previous studies indicated that the response of zirconia to plasma treatment varies significantly depending on the type of zirconia and bonding reagents used. Certain plasma treatments are detrimental to the bonding strength between the zirconia and bonding material. In our opinion, the CAP technique still has notable potential for intraoral application. Based on the limited amount of currently available data, we also suggest that further testing of helium-based CAP on different types of zirconia is worthwhile.

## 5. Other Ceramics and Ceramic-Containing Materials

Plasma treatment has also been applied to other assorted ceramic and ceramic-containing restorative materials, such as resin nanoceramics (RNC), glass-ceramics, alumina ceramics, and polymer-infiltrated ceramic network (PICN).

### 5.1. Resin Nanoceramics (RNC)

RNC consist of a resin/polymer matrix filled with nanoscale zirconia, silica, or barium particles [87,88]. A study by Adımcı et al. (in which no plasma gas was specified) reported a ~20% increase in SBS (which was excessively high at 46.91 MPa) post plasma activation accompanied by a moderate increase in the incidence of mixed failure rather than adhesive failure [49] (Table 7). In contrast, a recent study showed that treatment of RNC with argon- or helium-CAP with or without HF modification provided orthodontically acceptable SBS values [89]. Argon-CAP displayed a slightly better improvement compared to helium-CAP [89], while additional HF treatment did not meaningfully improve the bonding strength or alter the incidence of failure mode [89]. Since HF is toxic and can potentially harm the patient [1,5], HF application does not seem to be necessary in preparing RNC ceramic restorations for orthodontic treatment if plasma treatment, specifically CAP, can be used.

### 5.2. Glass-Ceramics

Glass-ceramics are another type of ceramic material used in restorative and aesthetic dentistry [6] that are generally brittle [90] but have good oral biocompatibility and aesthetic qualities [91]. One type of glass-ceramic material, known as silicate glass-ceramic, consists of lithium silicate or lithium disilicate crystals in a glass matrix [6,91]. A few studies evaluated the effects of plasma surface treatment on silicate glass-ceramics’ SBS. For example, Lanza et al. utilized argon and oxygen gases for CAP treatment of lithium silicate ceramic in combination with a variety of primers [41]. Although the SBS values provided by CAP treatment alone were very low, applying HF post-CAP treatment significantly increased the SBS of the lithium silicate ceramics [41]. This trend was also validated by another study conducted by Alayad et al., in which the gas used for CAP treatment was not specified: the plasma-treated group likewise had a much lower SBS value than the HF-treated non-plasma group [92]. However, there was marked variation among post-CAP treatment SBS values, seemingly dependent on the type of primer used, in the study of Alayad et al.

Interestingly, Bitencourt et al. reported that CAP treatment of lithium disilicate glass-ceramic resulted in a higher SBS than the HF treatment when argon, methane, and HMDSO gases were used, but not when argon, oxygen, and HMDSO gases were used [93]. However, after thermocycling, the SBS value of argon/methane/HMDSO-CAP treated lithium disilicate glass-ceramic was significantly lower than that of the HF-treated one [93]. Moreover, the overly long treatment time of 30 min and the toxicity of HMDSO make the argon/methane/HMDSO-CAP method described by Bitencourt et al. [93] unsuitable for chairside usage. Thus, no study has reported a plasma modification method that can be used intraorally to provide glass-ceramics with a better SBS than HF application does (Table 8).

### 5.3. Alumina Ceramics

Only one study has been published regarding the effect of plasma surface treatment on the SBS of alumina ceramic; it reported that radiofrequency plasma spraying resulted in an SBS value of 15.2 MPa, acceptable for orthodontic treatment [94] (Table 9). Despite the fact that the treatment time was not specified, the treatment’s usage of toxic HMDSO gas and radiofrequency plasma spray [94] prevent this technology from being used intraorally.

### 5.4. Polymer-Infiltrated Ceramic Network (PICN)

Likewise, only one published manuscript described the effect of plasma on PICN, a dental restorative material in which the ceramic component consists of feldspathic ceramic and alumina [95]. Here, the 30 s (short treatment time), argon-alone CAP application decreased the SBS values to half of those of the respective controls, regardless of the types of bonding reagents used [95] (Table 10), indicating that this treatment is not effective.

## 6. Discussion

Finding a plasma treatment that can consistently improve bonding strength and be delivered chairside safely and easily would improve orthodontic clinical care. Of all the types of plasma treatment that have been evaluated thus far, argon CAP is the most investigated; however, outcomes are inconsistent when different ceramics are used as the substrate. In addition, the choice of primer and bonding reagent also influences the effects of argon CAP. Thus, the feasibility of argon CAP’s usage in a clinician- and chairside-friendly surface conditioning treatment is considerably reduced because its effectiveness depends on the type of ceramic, primer, and bonding reagent used and it is difficult for clinicians to identify the specific type of ceramic used after restorations are delivered intraorally. On the other hand, based on the limited available evidence discussed above, helium CAP treatment holds great potential due to its usage of a non-toxic gas, short treatment time, and significant and consistent improvement of SBS among porcelain, zirconia, and RNC surfaces.

There is no doubt that this review has certain limitations. Firstly, the majority of the articles included and discussed in the current review were not tested in an orthodontic setting (i.e., with the bonding of brackets), and all of them were conducted in an in vitro environment. In addition, most of the studies used new ceramic blocks/plates that may not represent the surface condition of restorations that have been in the oral cavity for a period of time. Meanwhile, it is well known that the properties of the substance ceramics are contingent on their chemical compositions, which is particularly true for zirconia materials that can be categorized into distinct variants. Unfortunately, detailed information regarding the zirconia types (e.g., the yttria stabilizer content) is not always available in the reviewed manuscripts, which prohibits further comparison between different studies, particularly in the sense of underlying mechanisms and influence on the surface chemical composition. Therefore, a significant amount of research must be conducted before plasma treatment of restorative and aesthetic ceramic materials can be used for orthodontic purposes.

## 7. Conclusions

Based on the currently available literature, in our opinion, cold atmospheric plasma (CAP) treatment using helium might be a potential treatment method for porcelain, zirconia, and other types of ceramic. More importantly, CAP treatment using helium has the potential to be carried out chairside due to its non-toxicity, low temperature, and short treatment time. However, because all the available studies were conducted in vitro and not tested in an orthodontic setting, further research must be conducted to ascertain the effectiveness of specific plasma treatments relative to current orthodontic bonding treatments in vivo.

## Figures and Tables

**Table 1:** SBS and failure mode of plasma-treated feldspathic porcelain. CAP: cold atmospheric plasma; SBS: shear bond strength; TEGDMA: triethylene glycol dimethacrylate; HMDSO: hexamethyldisiloxane. For failure mode, A: adhesive failure; C: cohesive failure; M: mixed failure.

Plasma Type	Plasma Gas(es)	Primer (Y/N)	Subgroup Differences		SBS (MPa)	% of Control SBS	Failure Mode (A/C/M)	Control Failure Mode (A/C/M)	Ref.
CAP	Not specified	Y	-		43.16 ± 8.56	119.96%	20/20/60	30/30/40	[49]
CAP	Helium, TEGDMA	N	Voltage	9 V	25.8 ± 7.1	160.25%	35/30/35	100/0/0	[50]
				15 V	26.6 ± 7.4	165.22%	30/35/35		
				18 V	29.5 ± 9.3	183.23%	20/35/45		
CAP	Helium, water	N	Gas type		26.3 ± 6.3	129.56%	60/10/30	100/0/0	[51]
	Helium, HMDSO				11.5 ± 2.7	56.65%	100/0/0		
	Helium, benzene				28.4 ± 5.4	139.90%	70/25/5		
	Helium, HMDSO, benzene				32.4 ± 3.5	159.61%	50/45/5		
CAP	Helium, TEGDMA	N	Gas type		14.8 ± 3.7	128.70%	70/0/30	90/0/10	[52]
	Helium, TEGDMA, water				20.0 ± 3.9	173.91%	30/0/70		
	Helium, TEGDMA, water, HMDSO				14.7 ± 4.0	127.83%	100/0/0		
Etching	Oxygen	Y	-		72.83 ± 16.02	140.49%	-	-	[53]

**Table 3:** SBS and failure mode of zirconia ceramics treated with PECVD. PECVD: plasma-enhanced chemical vapor deposition; SBS: shear bond strength; HMDSO: hexamethyldisiloxane; TMS: tetramethylsilane. For failure mode, A: adhesive failure; C: cohesive failure; M: mixed failure.

Plasma Gas(es)	Zirconia Type	Primer (Y/N)	Subgroup Differences		SBS (MPa)	% of Control SBS	Failure Mode (A/C/M)	Control Failure Mode (A/C/M)	Ref.
Argon, oxygen, HMDSO	Y-TZP cubes (Vita Zahnfabrik, Bad Säckingen, Germany)	Y	not thermocycled		3.6 ± 1.0	39.13%	100/0/0	80/0/20	[25]
			thermocycled		-	-	-	90/0/10	
Argon, oxygen, hydrogen, sulfur hexafluoride			not thermocycled		11.1 ± 3.6	120.65%	100/0/0	80/0/20	
			thermocycled		3.8 ± 0.7	60.32%	100/0/0	90/0/10	
Argon, oxygen, HMDSO	Cercon Y-TZP blocks (Dentsply, Charlotte, NC, USA)	Y	-		0.45	2.36%	-	-	[72]
Argon, hydrogen, TMS	Cercon base block (DeguDent, Madrid, Spain)	Y	-		10.3 ± 2.8	228.89%	-	-	[73]
Argon, hydrogen, benzene					10.1 ± 4.1	224.44%	-	-	
Argon, hydrogen, TMS, benzene					22.7 ± 3.7	504.44%	-	-	
SiH4 (silane)	VITA YZ HT blocks (Vita Zahnfabrik, Bad Säckingen, Germany)	Y	not thermocycled	plasma treated for 30 s	24.8 ± 5.0	166.44%	-	-	[74]
				plasma treated for 60 s	22.1 ± 8.5	148.32%			
				plasma treated for 120 s	23.0 ± 2.5	154.36%			
				plasma treated for 300 s	20.1 ± 6.4	134.90%			
			thermocycled	plasma treated for 30 s	3.9 ± 0.7	390.00%	-	-	
				plasma treated for 60 s	3.6 ± 0.8	360.00%			
				plasma treated for 120 s	6.1 ± 1.8	610.00%			
				plasma treated for 300 s	5.6 ± 1.8	560.00%			

**Table 4:** SBS and failure mode of zirconia ceramic treated with plasma fluorination. SBS: shear bond strength. For failure mode, A: adhesive failure; C: cohesive failure; M: mixed failure.

Plasma Gas(es)	Zirconia Type	Primer (Y/N)	SBS (MPa)	% of Control SBS	Failure Mode (A/C/M)	Control Failure Mode (A/C/M)	Ref.
Sulfur Hexafluoride	ZircCAD blocks (Ivoclar Vivadent, Schaan, Liechtenstein)	Y	26.3 ± 6.4	260.40%	30/0/70	100/0/0	[78]
Sulfur Hexafluoride	Lava plates and cylinders (3M ESPE AG, St. Paul, MN, USA)	N	26.7 ± 4.9	290.22%	100/0/0	100/0/0	[79]
Sulfur Hexafluoride	Lava plates (3M ESPE AG, St. Paul, MN, USA)	N	37.3 ± 4.6	405.43%	40/60/0	100/0/0	[80]

**Table 5:** SBS and failure mode of zirconia ceramic treated with magnetron sputtering. SBS: shear bond strength. For failure mode, A: adhesive failure; C: cohesive failure; M: mixed failure.

Plasma Gas(es)	Zirconia Type	Primer (Y/N)	Subgroup Differences	SBS (MPa)	% of Control SBS	Failure Mode (A/C/M)	Control Failure Mode (A/C/M)	Ref.
Argon	ICE Zirkon blocks (Zirkonzahn, Gais BZ, Italy)	N	plasma treated for 2.5 min	12.15 ± 2.34	70.97%	-	-	[81]
			plasma treated for 15 min	14.52 ± 4.50	84.81%	-	-	
			plasma treated for 20 min	24.69 ± 5.08	144.22%	-	-	
		Y	plasma treated for 2.5 min	21.22 ± 3.91	110.06%	-	-	
			plasma treated for 15 min	24.41 ± 4.55	126.61%	-	-	
			plasma treated for 20 min	28.45 ± 2.41	147.56%	-	-	
Argon, oxygen	yttria-stabilized zirconia (Tosoh, Tokyo, Japan)	Y	-	30.86 ± 3.52	333.26%	-	-	[82]
Argon, oxygen	-	Y	-	32.8 ± 5.4	285.22%	-	-	[83]

**Table 6:** SBS and failure mode of zirconia ceramic treated with other types of plasma treatment. SBS: shear bond strength; RF: radiofrequency; HMDSO: hexamethyldisiloxane. For failure mode, A: adhesive failure; C: cohesive failure; M: mixed failure.

Plasma Type	Plasma Gas(es)	Zirconia Type	Primer (Y/N)	Subgroup Differences		SBS (MPa)	% of Control SBS	Failure Mode (A/C/M)	Control Failure Mode (A/C/M)	Ref.
Etching	Oxygen	Talent dental discs (FP50-XP)	Y	-		17.8 ± 2.1	291.80%	0/30/70	30/0/70	[84]
Silica coating	Argon	Talent dental discs (FP50-XP	Y	-		19.6 ± 2.6	321.31%	0/70/30	30/0/70	[84]
RF plasma spraying	Oxygen, HMDSO		N	hot isostatic pressed yttrium-oxide partially stabilized zirconia blocks (Nobel Biocare)		5.3 ± 0.7	353.33%	-	-	[24]
				glossy dense zirconia blocks (Zircar Zirconia, Inc.)		3.5 ± 0.7	437.5%	-	-	
Glow-discharge plasma	Not specified	NANOZR disks (Panasonic Health Care)	N	Clearfil SA Luting Plus	not thermocycled	24.6 ± 3.7	153.75%	0/0/100	0/0/100	[85]
					thermocycled	16.3 ± 2.6	136.97%	0/0/100	50/0/50	
				luting composite of Panavia V5 without primer	not thermocycled	4.9 ± 2.2	132.43%	100/0/0	100/0/0	
					thermocycled	2.4 ± 0.2	104.35%	100/0/0	100/0/0	
Irradiation	Not specified	TZ-3YB-E discs (Tosoh, Osaka, Japan)	N	Clearfil Protect Bond		7.96 ± 2.76	93.76%	-	-	[86]
				Clearfil S^3^ Bond Plus		11.58 ± 2.69	114.09%			

**Table 7:** SBS and failure mode of plasma-treated resin nanoceramic. CAP: cold atmospheric plasma. SBS: shear bond strength. For failure mode, A: adhesive failure; C: cohesive failure; M: mixed failure.

Plasma Type	Plasma Gas(es)	Primer (Y)	Subgroup Differences		SBS (MPa)	% of Control SBS	Failure Mode (A/C/M)	Control Failure Mode (A/C/M)	Ref.
CAP	Not specified	Y	-		46.91 ± 4.33	120.75%	10/20/70	20/30/50	[49]
CAP	Argon	N	no HF	not thermocycled	33.97 ± 2.04	201.84%	20/0/80	100/0/0	[89]
				thermocycled	28.28 ± 2.56	211.36%	40/0/60	100/0/0	
		Y	with HF	not thermocycled	33.78 ± 1.60	159.57%	20/0/80	30/0/70	
				thermocycled	32.38 ± 1.42	185.35%	30/0/70	50/0/50	
	Helium	N	no HF	not thermocycled	27.93 ± 1.74	165.95%	30/0/70	100/0/0	
				thermocycled	23.02 ± 2.62	172.05%	40/0/60	100/0/0	
		Y	with HF	not thermocycled	28.51 ± 1.71	134.67%	30/0/70	30/0/70	
				thermocycled	25.98 ± 1.74	148.71%	30/0/70	50/0/50	

**Table 8:** SBS and failure mode of CAP-treated glass ceramics. CAP: cold atmospheric plasma. SBS: shear bond strength. HMDSO: hexamethyldisiloxane. ACPS: acryloyloxypropyltrimethoxysilane; STYRX: styrylethyltrimethoxysilane; ALAP: 3-(N-allylamino) propyltrimethoxysilane; BATS: bis(2-hydroxyethil)–3-aminopropyltriethoxysilane; MBP: Monobond Plus, ZPP: ZPrimePlus. For failure mode, A: adhesive failure; C: cohesive failure; M: mixed failure. * Control group was treated with HF.

Plasma Gas(es)	Primer (Y/N)	Subgroup Differences		SBS (MPa)	% of Control SBS	Failure Mode (A/C/M)	Control Failure Mode (A/C/M)	Ref.
Argon, oxygen	N	No HF	short-term water storage	2.5 ± 2.5	71.43%	100/0/0	100/0/0	[41]
			long-term water storage	0.0 ± 0.0	-	100/0/0	-	
		With HF	short-term water storage	14.0 ± 1.4	153.85%	100/0/0	91.6/0.4/8.0	
			long-term water storage	5.1 ± 2.3	87.93%	100/0/0	91/0/9	
	ACPS	No HF	short-term water storage	11.6 ± 4.5	165.71%	83.3/0/16.7	0/100/0	
			long-term water storage	1.3 ± 1.0	76.47%	100/0/0	0/92/8	
		With HF	short-term water storage	28.7 ± 4.8	118.60%	0/100/0	58.3/16.7/25	
			long-term water storage	22.6 ± 5.1	105.61%	0/92/8	50/33/17	
	STYRX	No HF	short-term water storage	12.1 ± 4.0	295.12%	83.3/0/16.7	0/16.7/83.3	
			long-term water storage	2.8 ± 2.0	147.37%	100/0/0	0/75/25	
		With HF	short-term water storage	25.7 ± 4.9	120.66%	0/16.7/83.3	0/75/25	
			long-term water storage	19.1 ± 2.5	117.18%	0/75/25	83/0/17	
	ALAP	No HF	short-term water storage	2.4 ± 2.8	85.71%	100/0/0	100/0/0	
			long-term water storage	0.2 ± 0.6	285.71%	100/0/0	100/0/0	
		With HF	short-term water storage	15.1 ± 2.3	260.34%	100/0/0	90.9/0/9.1	
			long-term water storage	7.7 ± 1.8	256.67%	100/0/0	100/0/0	
	BATS	No HF	short-term water storage	2.7 ± 2.7	117.39%	100/0/0	100/0/0	
			long-term water storage	0.1 ± 0.3	-	100/0/0	100/0/0	
		With HF	short-term water storage	11.4 ± 3.0	120.00%	100/0/0	100/0/0	
			long-term water storage	3.7 ± 0.8	59.68%	100/0/0	100/0/0	
	MBP	No HF	short-term water storage	6.4 ± 2.4	106.67%	58.3/0.01/41.6	0/91.6/8.3	
			long-term water storage	0.4 ± 0.7	22.22%	100/0/0	8/59/33	
		With HF	short-term water storage	23.7 ± 5.8	139.41%	0/91.6/8.3	25/33.4/41.6	
			long-term water storage	20.2 ± 5.5	169.75%	8/59/33	58/8/33	
	ZPP	No HF	short-term water storage	2.6 ± 2.0	83.87%	100/0/0	100/0/0	
			long-term water storage	0.0 ± 0.0	-	-	100/0/0	
		With HF	short-term water storage	19.8 ± 3.9	131.13%	100/0/0	100/0/0	
			long-term water storage	14.2 ± 3.9	184.42%	100/0/0	100/0/0	
Not specified	Y	-		14.28 ± 0.62	66.48% *	80/10/10	20/70/10 *	[92]
Argon, methane, HMDSO	Y	not thermocycled		10.44	143.01% *	100/0/0	80/0/20 *	[93]
		thermocycled		1.9	27.90% *	100/0/0	100/0/0 *	
Argon, oxygen, HMDSO		not thermocycled		6.54	89.59% *	100/0/0	80/0/20 *	
		thermocycled		7.29	107.05% *	100/0/0	100/0/0 *	

**Table 9:** SBS and failure mode of plasma-treated alumina ceramic. CAP: cold atmospheric plasma. SBS: shear bond strength. HMDSO: hexamethyldisiloxane. For failure mode, A: adhesive failure; C: cohesive failure; M: mixed failure.

Plasma Type	Plasma Gas(es)	Primer (Y/N)	Subgroup Differences	SBS (MPa)	% of Control SBS	Failure Mode (A/C/M)	Control Failure Mode (A/C/M)	Ref.
RF plasma spraying	Oxygen, HMDSO	N	-	15.2 ± 2.6	214.08%	-	-	[94]

**Table 10:** SBS and failure mode of plasma-treated polymer-infiltrated ceramic network (PICN). CAP: cold atmospheric plasma. SBS: shear bond strength. For failure mode, A: adhesive failure; C: cohesive failure; M: mixed failure. * Control group was treated with HF.

Plasma Type	Plasma Gas(es)	Primer (Y/N)	Subgroup Differences		SBS (MPa)	% of Control SBS	Failure Mode (A/C/M)	Control Failure Mode (A/C/M)	Ref.
CAP	Argon	Y	Panavia V5	not thermocycled	5.8 ± 1.4	58.00% *	-	-	[95]
				thermocycled	2.9 ± 0.8	31.87% *	-	-	
			RelyX Ultimate	not thermocycled	10.1 ± 2.1	64.33% *	-	-	
				thermocycled	7.5 ± 1.7	63.56% *	-	-	

## Data Availability

The data presented in this study are contained within this article.
